# The complexity of mitochondrial outer membrane permeability and VDAC regulation by associated proteins

**DOI:** 10.1007/s10863-018-9765-9

**Published:** 2018-07-12

**Authors:** Aleksandr Klepinin, Lyudmila Ounpuu, Kati Mado, Laura Truu, Vladimir Chekulayev, Marju Puurand, Igor Shevchuk, Kersti Tepp, Anu Planken, Tuuli Kaambre

**Affiliations:** 1 Laboratory of Bioenergetics, National Institute of Chemical Physics and Biophysics, Akadeemia tee 23, 12618 Tallinn, Estonia; 2 Oncology and Hematology Clinic at the North Estonia Medical Centre, Tallinn, Estonia

**Keywords:** Mitochondria, Adenylate kinase, Glycolysis, OXPHOS, Tubulin, Warburg effect

## Abstract

Previous studies have shown that class II β-tubulin plays a key role in the regulation of oxidative phosphorylation (OXPHOS) in some highly differentiated cells, but its role in malignant cells has remained unclear. To clarify these aspects, we compared the bioenergetic properties of HL-1 murine sarcoma cells, murine neuroblastoma cells (uN2a) and retinoic acid - differentiated N2a cells (dN2a). We examined the expression and possible co-localization of mitochondrial voltage dependent anion channel (VDAC) with hexokinase-2 (HK-2) and βII-tubulin, the role of depolymerized βII-tubuline and the effect of both proteins in the regulation of mitochondrial outer membrane (MOM) permeability. Our data demonstrate that neuroblastoma and sarcoma cells are prone to aerobic glycolysis, which is partially mediated by the presence of VDAC bound HK-2. Microtubule destabilizing (colchicine) and stabilizing (taxol) agents do not affect the MOM permeability for ADP in N2a and HL-1 cells. The obtained results show that βII-tubulin does not regulate the MOM permeability for adenine nucleotides in these cells. HL-1 and NB cells display comparable rates of ADP-activated respiration. It was also found that differentiation enhances the involvement of OXPHOS in N2a cells due to the rise in their mitochondrial reserve capacity. Our data support the view that the alteration of mitochondrial affinity for ADNs is one of the characteristic features of cancer cells. It can be concluded that the binding sites for tubulin and hexokinase within the large intermembrane protein supercomplex Mitochondrial Interactosome, could be different between muscle and cancer cells.

**Electronic supplementary material:**

The online version of this article (10.1007/s10863-018-9765-9) contains supplementary material, which is available to authorized users.

## Introduction

Malignant transformation of cells leads to reprogramming in numerous signaling and metabolic pathways, especially in regard to energy metabolism. Targeting of tumor-initiating and cancer cell energy metabolism has been proposed to be a novel and highly effective strategy for the selective ablation of malignant tumors (Aminzadeh et al. 2015; Gogvadze et al. 2009; Lamb et al. 2015; Moreno-Sanchez et al. 2007).

Recently, it was demonstrated that the mitochondrial outer membrane (MOM) voltage-dependent anion channel (VDAC) is the main switch between mitochondrial oxidative phosphorylation (OXPHOS) and glycolysis in malignant cells and it chould be a good target for a new generation of cancer therapy (Carre et al. 2002; Maldonado 2017). Mitochondrial VDAC plays a key role in maintaining high rates of OXPHOS as well as in the realization of apoptotic programs (Shoshan-Barmatz et al. 2006, 2009, 2017). It was reported that in brain and tumor cells, some hexokinase isoforms can bind to the VDAC in the MOM thereby suppressing cytochrome c release and apoptotic cell death (Arzoine et al. 2009). This channel is involved in the transport of respiratory substrates, Ca^2+^, ATP, ADP and inorganic phosphate across the external mitochondrial membranes supporting the high efficiency of OXPHOS and the Krebs cycle (Noskov et al. 2013; Rostovtseva and Colombini 1997; Shoshan-Barmatz et al. 2018).

Studies performed during the past decade have shown that in mammalian cells the permeability of mitochondrial VDAC towards adenine nucleotides (AND(s)) and respiratory substrates is a precisely controlled process (Rostovtseva and Bezrukov 2012). But, the precise regulatory factors mediating this VDAC permeability, especially, in cancer cells, are studied insufficiently. The regulation of MOM permeability has been quite thoroughly studied on heart and skeletal muscles. These in situ studies have shown thatin slow-twitch skeletal and heart muscles the value of apparent Michaelis - Menten constant Km for ADP is high (there exist diffusion obstacles for adenine nucleotides (ADNs), but for fast-twitch skeletal muscles the permeability of MOM for ADNs is normally high and the value of the Michaelis-Menten constant is 20 times lower than is oxidative muscles (Kuznetsov et al. 1996; Saks et al. 1995; Saks and Aliev 1996; Saks et al. 1994, 1998). Ultrastructural studies have revealed multiple connections between cytoskeletal elements and mitochondria in different types of cells. Several candidate proteins that can regulate the MOM permeability for AND(s) have been proposed like the α-β tubulin heterodimer (Guzun et al. 2015; Maldonado and Lemasters 2014; Maldonado et al. 2013; Rostovtseva and Bezrukov 2012; Saks et al. 2010), desmin (Appaix et al. 2003; Capetanaki et al. 2007; Guzun et al. 2012; Saetersdal et al. 1990; Winter et al. 2015), plectin (Reipert et al. 1999; Winter et al. 2008, 2015), α-synuclein (Hoogerheide et al. 2017; Rostovtseva et al. 2015; Shen et al. 2014; Zhang et al. 2016), αβ-crystallin (Diokmetzidou et al. 2016), microtubule-associated proteins (Guzun et al. 2012), and some hexokinase (HK) isoforms (Beutner et al. 1996; Bryan and Raisch 2015; Lemeshko 2014; Lu et al. 2015; Mathupala et al. 2009; Nederlof et al. 2014).

In cells with a high energy demand the OXPHOS system is organized into large protein complexes, one of them is the protein supercomplex Mitochondrial Interactosome (MI) (Guzun et al. 2012; Saks et al. 2010; Tepp et al. 2011; Timohhina et al. 2009). MI is a large transmembrane complex consisting of ATP - synthasome, mitochondrial creatine kinase (MtCK) or other representatives of mitochondrial kinases, VDAC, and some protein factors, which regulate the MOM permeability for adenine nucleotides. It has been demonstrated that in rat heart cardiomyocytes (CMs) βII-tubulin binds to VDAC regulating the permeability of this mitochondrial channel for adenine nucleotides and promoting thereby the generation of phosphocreatine (PCr) via MtCK (Guzun et al. 2015; Timohhina et al. 2009). It was found, that during carcinogenesis the composition and structure of MI may be radically reorganized due to profound alterations in the expression of its components (Chevrollier et al. 2011; Koit et al. 2017).

Two mechanisms by which the MOM permeability is regulated in cancer cells have been proposed. First, according to the model proposed by Pedersen and co-workers, the interaction of VDAC with HK-2 is one of the main pathways mediating the “Warburg effect” or aerobic glycolysis in cancer cells (Mathupala et al. 2009; Pedersen 2007b). It has been shown that HK-2 binding on VDAC channel keeps it in an open state (Majewski et al. 2004) and allows the HK-2 to use intra-mitochondrially generated ATP to phosphorylate glucose (Cesar Mde and Wilson 1998). The second mechanism proposed by Maldonado and co-workers, demonstrates that in hepatocarcinoma cells VDAC is blocked by free tubulin which induces malignant cells to switch to aerobic glycolysis (Maldonado et al. 2010). They have demonstrated that if the level of non-polymerized α-β heterodimer tubulin increases in liver cancer cells, it leads to rising of mitochondrial membrane potential, which induces closing of VDAC. Recently, our study on rat muscle tissues, suggested that only non-polymerized βII-tubulin in heart and soleus muscles plays an important role in the regulation of MOM permeability for ADP (Varikmaa et al. 2014). In both studies the free dimeric tubulin has been shown to affect VDAC permeability, but its effect depends on polymerized/dimeric tubulin ratio.

In the current study we therefore hypothesized that in cancer cells the free βII-tubulin can compete with HK-1 or HK-2 for the binding sites on VDAC(s) consequently, in order to regulate the aerobic glycolytics in tumor cells. The aim of the present study was to clarify the role of free/polymerized βII-tubulin and HK-2 in regulation of energy transfer in malignant cells of different histological origin. For this purpose, experiments were performed on Warburg phenotype cell lines, such as undifferentiated murine neuroblastoma cells (N2a) and retinoic acid (RA)-differentiated NB cells, as well as on HL-1 cardiac sarcoma cells, where free/polymerized level was regulated by the tubulin depolymerizing agent colchicine and tubulin polymerizing agent taxol (See Graphical Abstract in Supplementary material).

## Materials and methods

### Chemicals

Dulbecco’s Modified Eagle Medium (DMEM) and phosphate buffered saline (PBS, Ca/Mg free) were obtained from Corning, Inc. (USA) whereas heat-inactivated fetal bovine serum (FBS), accutase, penicillin-streptomycin solution (100×), gentamicin and 0.05% Trypsin-EDTA were purchased from Gibco Life Technologies (Grand Island, NY, USA). Primary and secondary antibodies were obtained from Santa Cruz Biotechnology Inc. (USA) or Abcam PLC (UK), rabbit polyclonal antibodies against VDAC1 kindly donated by Dr. Catherine Brenner from Paris-Sud University, France. Unless otherwise stated, all other chemicals were purchased from Sigma-Aldrich Company (St. Louis, USA).

### Cultivation of murine neuroblastoma (Neuro-2a) cells and their differentiation

The stock culture of N2a cells was obtained from the American Type Culture Collection (ATCC, Cat. No. CCL-131). These NB cells were grown in T75 flaks (Greiner bio-one) as a loosely adhering monolayer at 37 °C in 5% CO_2_ in a high glucose (4.5 g/l) DMEM supplemented with L-glutamine, 10% FBS, 100 U/ml penicillin, 100 μg/ml streptomycin, and 50 μg/ml gentamicin. The neural differentiation of N2a cells to cholinergic neurons was induced by their cultivation with 10 μM all-trans-retinoic acid (RA) in a complete growth medium, but at a decreased (1%) concentration of FBS for seven days (Blanco et al. 2001; Klepinin et al. 2014).

### Cultivation of HL-1 tumor cells

The non-beating HL-1 cell line derived from tumoral atrial cardiac myocytes of mice (Claycomb et al. 1998; Pelloux et al. 2006) was used. These tumor cells were kindly provided by Dr. Andrey V. Kuznetsov (Innsbruck Medical University, Austria). HL-1 cells were grown in fibronectin gelatin coated (5 μg/ml and 0.2%, respectively) T75 flasks containing Claycomb medium (Sigma-Aldrich) supplemented with 10% FBS, 100 U/ml penicillin, 100 μg/ml streptomycin, 50 μg/ml gentamicin, 2 mM L-glutamine, 0.1 mM norepinephrine, and 0.3 mM ascorbic acid.

### Cell viability and proliferation assays

The number of viable cells was estimated by trypan blue exclusion assay, while the rate of cell proliferation by MTT assay as described in our prior work (Klepinin et al. 2014).

### Cell permeabilization and measurements of OXPHOS function in cells

To examine the functional capacity of mitochondria in N2a and HL-1 tumor cells, we applied the permeabilized cell technique developed by Kuznetsov and colleagues (Kuznetsov et al. 2008). This method allows to studying the function of mitochondria in situ in tissues and cells without isolation of these organelles. The permeabilization procedure leaves intact intracellular interactions of mitochondria with cytoskeleton and other organelles.

Plasma membranes were permeabilized with saponin at 40 μg/ml (N2a cells) or digitonin at 25 μg/ml treatments (for HL-1 cells). The rate of O_2_ consumption in permeabilized N2a or HL-1 cells was measured at 25 °C with an Oxygraph-2 K respirometer (Oroboros Instruments, Austria) in respiration medium-B (Kuznetsov et al. 2008) supplemented with 5 mM glutamate, 2 mM malate and 10 mM succinate as respiratory substrates; the solubility of oxygen was taken as 240 nmol/ml (Gnaiger 2001). For determination of the reserve respiratory capacity of mitochondria, the rate of cellular O_2_ consumption was measured before and after a stepwise addition of the mitochondrial uncoupler – carbonyl cyanide p-(trifluoro-methoxy)phenyl-hydrazone (FCCP). The rates of O_2_ consumption were normalized per mg cellular protein. The protein concentration in cell lysates was determined using the Pierce BCA Protein Kit.

### Determination of apparent Michaelis-Menten constant values for exogenously added ADP

The apparent Km and Vm values for exogenously added ADP (^ADP^K_m_) were calculated from ADP titration experiments using the corresponding non-linear regression equation.

### Analysis of OXPHOS coupling with hexokinase (HK)-mediated processes

The coupling between mitochondrially bound HK(s) and the OXPHOS system in permeabilized cells was assayed by oxygraphy, through stimulation of mitochondrial respiration by locally generated ADP as described earlier (Eimre et al. 2008; Kaldma et al. 2014). The effect of glucose on mitochondrial respiration was expressed by the glucose index (I_GLU_) that was calculated according to the equation I_GLU_ (%) = [(V_GLU_ - V_ATP_)/(V_ADP_ - V_ATP_)]*100, where V_ADP_ is the rate of O_2_ consumption in the presence of 2 mM ADP, V_GLU_ is the respiration rate with 10 mM glucose and V_ATP_ is respiration rate with 0.1 mM ATP; i.e. this index reflects the degree of glucose-mediated stimulation of mitochondrial respiration as compared with the maximal ADP-activated rate of O_2_ consumption.

### Immunofluorescence analysis

Immunocytochemistry along with confocal microscopy imaging were applied to visualize the expression and possible colocalization of VDAC, with HK-2, and βII-tubulin in HL-1 and N2a cells. For immunofluorescence studies the following primary antibodies were used: rabbit polyclonal antibodies vs. VDAC1 (kindly provided by Dr. Catherine Brenner; Paris-Sud University, Paris, France), goat polyclonal antibodies vs. HK-2 (sc-6521; Santa Cruz Biotechnology, Inc., USA), and mouse monoclonal antibody to TUBB2A (ab92857; Abcam®, UK). After overnight incubation (at 4 °C) with the indicated primary antibodies, HL-1 cells were washed with a 2% BSA solution and co-incubated with the following secondary fluorescent antibodies: a) anti-rabbit IgG labeled with DyLight-488 (ab96895) giving green fluorescence, to visualize VDAC; b) anti-goat Cy-3 labeled IgG that gives red florescence, to stain HK-2; and c) donkey anti-mouse IgG-CFL: 647 sc-362,288 (violet color) or goat anti-mouse DyLight-550 labeled IgG (ab96880) yielding red, to stain βII-tubulin. ProLong Gold antifade reagent supplemented with 4′,6-diamidino-2-phenylindole dihydrochloride (DAPI, Molecular Probes™) used for visualizing the cell nucleus. The cells were then imaged by an Olympus FluoView FV10i-W inverted laser scanning confocal microscope. For immunofluorescent studies, N2a cells were seeded in 12-well plates (at a density of 1 × 10^4^ cells/well) over glass coverslips, treated or not with 10 μM RA, and then immunostained mostly as described above for HL-1 cells. The presence of mitochondria in N2a cells was also estimated through the selective labeling of the VDAC1 (sc-8828, Santa Cruz Biotechnology, Inc., USA). The visualization of VDAC1 protein expression was carried out using fluorescent secondary donkey anti-goat Cy-3 (ab97115) antibodies (red fluorescence) and to visualize βII-tubulin donkey anti-rabbit IgG (Alexa Fluor® 488, ab150073, green fluorescence).

### SDS-PAGE and western blot analysis of the levels of beta-tubulin isotypes in N2a and HL-1 cells

The cells were washed twice with Ca/Mg-free PBS and then treated with a microtubule lysing buffer consisting of 100 mM PIPES, 5 mM MgCl_2_, 1 mM EGTA, 30% glycerol, 0.1% IGEPAL, 0.1% Tween-20, 0.1% Triton X-100, 0.1% beta-mercaptoethanol, 1 mM ATP, 0.1 mM GTP and a complete protease inhibitor cocktail (Roche); the recipe is according to Cytoskeleton, Inc. (USA). The lysate was homogenized by Retsch Mixer Mill at 25 Hz for 2 min, and incubated for 30 min at 35 °C. The obtained cell lysates were clarified by centrifugation at 21000 x *g* for 40 min at 35 °C. The protein concentration in lysates was determined using the Pierce BCA Protein Kit. Proteins were separated by 12% SDS-PAGE and transferred onto the PVDF membrane by Trans-Blot Semi-Dry Transfer system (Bio-Rad, Inc., USA).

To determine the presence of beta-tubulin isotypes Abcam mono- and polyclonal antibodies (anti-beta I Tubulin (ab11312), anti-Tubb2A (ab170931) and anti-beta III Tubulin (ab52901) were used. After the chemiluminescence reaction, the PVDF membranes were stained with Coomassie brilliant blue R250 to measure the total protein amount. The tubulin signal intensity was normalized against total protein intensities obtained from Coomassie staining. Quantification was performed by ImageJ software.

### Evaluation of soluble and polymerized beta-tubulins

The content of free and polymerized tubulin in HL-1 and N2a cells was assessed using a “Microtubules/Tubulin in vivo Assay “kit (Cytoskeleton Inc.) in accordance with the manufacturer’s manual. Cells were homogenized in cell lysis and microtubule stabilization buffer (100 mM PIPES pH 6.9, 5 mM MgCl_2_, 1 mM EGTA, 30% (*v*/v) glycerol, 0.1% Nonidet P40, 0.1% Triton X-100, 0.1% β-mercaptoethanol, 0.001% antifoam) supplemented with 0.1 mM GTP, 1 mM ATP and protease inhibitor cocktail. In addition, cell fractions containing 10 μM taxol and 2 mM CaCl_2_ were used as the positive and negative controls. Lysates were centrifuged at 2000 x *g* for 5 min at 37 °C to remove intact cells. Supernatants were centrifuged at 100000 x *g* for 30 min at 37 °C to separate microtubules from soluble (free) tubulin. The pellets containing polymerized tubulin were suspended in ice-cold 2 mM CaCl_2_.

Free tubulin and polymerized tubulin fractions were loaded on 10% polyacrylamide gels. Proteins were transferred using the Trans-Blot SD Semi-Dry Transfer Cell (BioRad). Blots were blocked in 5% nonfat milk and probed with anti Tubb2A (ab170931) antibody for 2 h at room temperature. Immunoblots were incubated with secondary antibodies (anti-mouse IgG, HRP, Abcam) for 1 h at room temperature. Detection was conducted using a chemiluminescence kit (Pierce ECL Western Blotting Substrate).

### Assessment of basic OXPHOS parameters in HL-1 and N2a cells pretreated with colchicine and taxol

Unless otherwise specified, these tumor cells were treated with colchicine (10 μM), taxol (10 μM) or DMSO (control) for 24 h at 37 °C. In some experiments, the influence of colchicine and taxol on the affinity of mitochondria to exogenously added ADP as well as their respiratory reserve capacity was also examined after a short-term (for 20 min) exposure of tumor cells to these microtubular toxins. (Maldonado et al. 2010). The following OXPHOS parameters were then assessed: basal respiration, ATP-linked respiration, proton leak, maximal respiration and mitochondrial reserve capacity (Supplementary Fig. 3; Fig. 5). Basal respiration was measured in medium-B supplemented with 5 mM glutamate, 2 mM malate and 10 mM succinate. Then, oligomycin (2.5 μM) was added to inhibit proton flow through ATP synthase blocking ATP-linked oxygen consumption. Maximal respiration was measured by exposing cells to carbonyl cyanide-p-trifluoromethoxyphenyl-hydrazon (FCCP), which uncoupled respiration from ATP production. In the presence of FCCP, respiration increased beyond the basal respiration by reserve capacity of mitochondria. Finally, the electron transport was inhibited by 10 μM antimycin, a complex III inhibitor, indicating the non-mitochondrial oxygen consumption. Proton leak was calculated by subtracting the rate of non-mitochondrial respiration from respiration that remained after ATP-synthase inhibition. The maximal respiration capacity was calculated by subtracting non-mitochondrial respiration rates from the FCCP induced maximal respiration. Changes in the ATP-linked respiration, proton leak, maximal respiration and reserve capacity were expressed as a percentage of basal respiration.

### Statistical analysis

All data points are presented as means ± standard error (SEM) from at least five separate experiments performed in duplicate. The statistical differences between the groups were calculated by the two-tailed Student’s t-test. Differences were considered to be statistically significant when *p* < 0.05.

## Results

### The effect of saponin/digitonin treatment on the intactness of mitochondrial membranes in N2a and HL-1 cells

The mitochondrial respiration in all studied permeabilized cell types was activated with 2 mM ADP and the rate of O_2_ consumption was increased by about 3–4 times (Supplementary Fig. 1). The subsequent addition of cytochrome c (Cyt c) to permeabilized cells did not cause an increase of more than 10% in the rate of oxygen consumption, which indicated the intactness of the outer mitochondrial membrane. After that, addition of carboxyatractyloside (CAT), an inhibitor of the adenine nucleotide translocator, decreased the respiration rate back to the basal level (V0), showing the intactness of the mitochondrial inner membrane. Experiments showed that all used cell cultures had similar rates of basal and State III respiration. Respiratory control index (RCI) values for uN2a, dN2a and HL-1 cells were calculated as 4.51 ± 0.63, 4.43 ± 0.22 and 5.11 ± 0.59, respectively.

We also showed that the permeabilization method does not affect the tubulin content in cell cultures (Supplementary Fig. 2).

### The intracellular content and distribution of tubulin in HL-1 and N2a cells

**Fig. 1:**
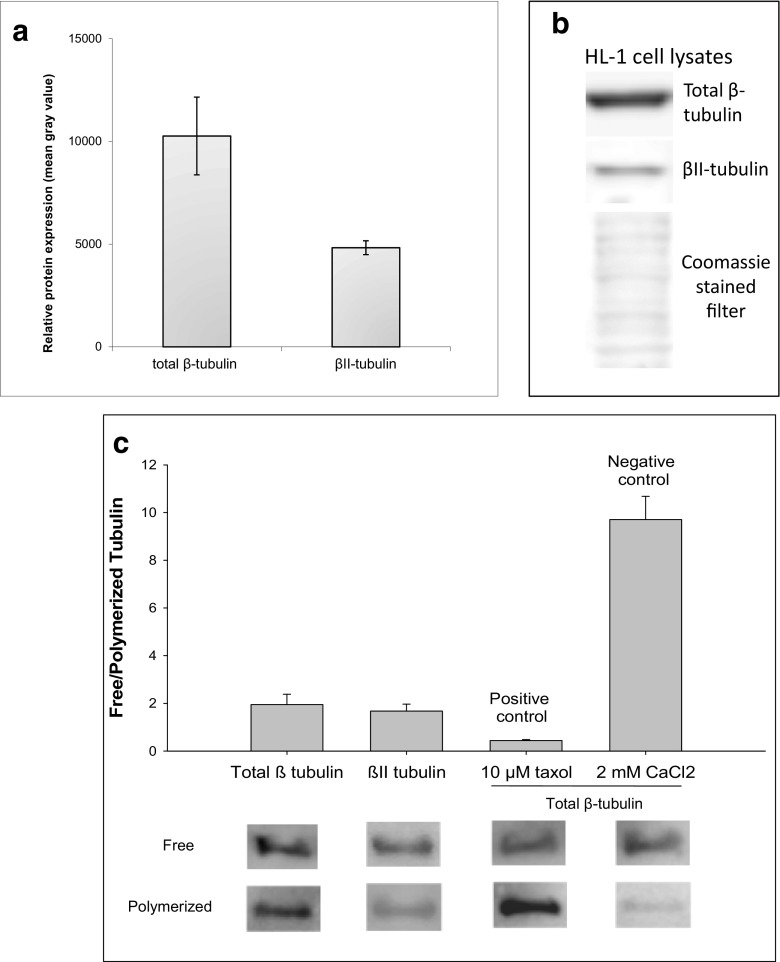
Western blot analysis for the presence of total β and βII-tubulin in HL-1 cells (**a**, **b**) as well as the levels of free and polymerized total β- and βII-tubulin in these tumor cell line (**c**); here, lower panel shows the representative immunoblot test for free and polymerized total β and βII tubulin in HL-1 cell. Upper panel shows a densitometric quantification of the total β and βII tubulin in the soluble and insoluble fractions of HL-1 cells. Error bars are the mean ± SE from 3 separate experiments; *p < 0.05 when compared to total β tubulin in HL-1 cells; **p < 0.005 when compared to βII-tubulin in HL-1 cells

**Fig. 2:**
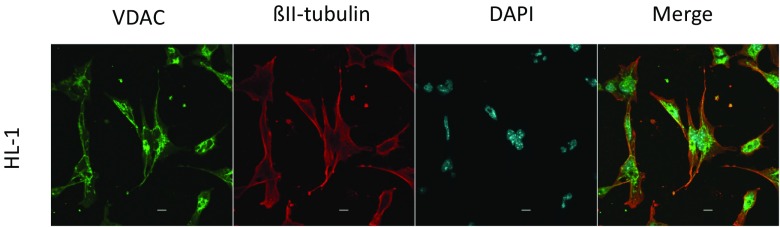
Confocal immunofluorescence imaging of the mitochondrial VDAC1 protein (green), βII-tubulin (red), nucleus (blue) and their colocalization in HL-1 tumor cells; bars are 10 μm

We checked total β and β-II tubulin expression in HL-1 cells. Our results showed that β-II tubulin constitutes about 50% of the total β-tubulin in those cells (Fig. 1a, b). Nevertheless, the amount of free/polymerized β-II tubulin and total β-tubulin was equal (Fig. 1c). Confocal microscopy showed, that a part of the mitochondria in HL-1 cells is distributed randomly, whereas other mitochondria are attached to βII-tubulin containing microfilaments, and concentrated around the cell nucleus - an area with an increased energy demand (Fig. 2).

**Fig. 3:**
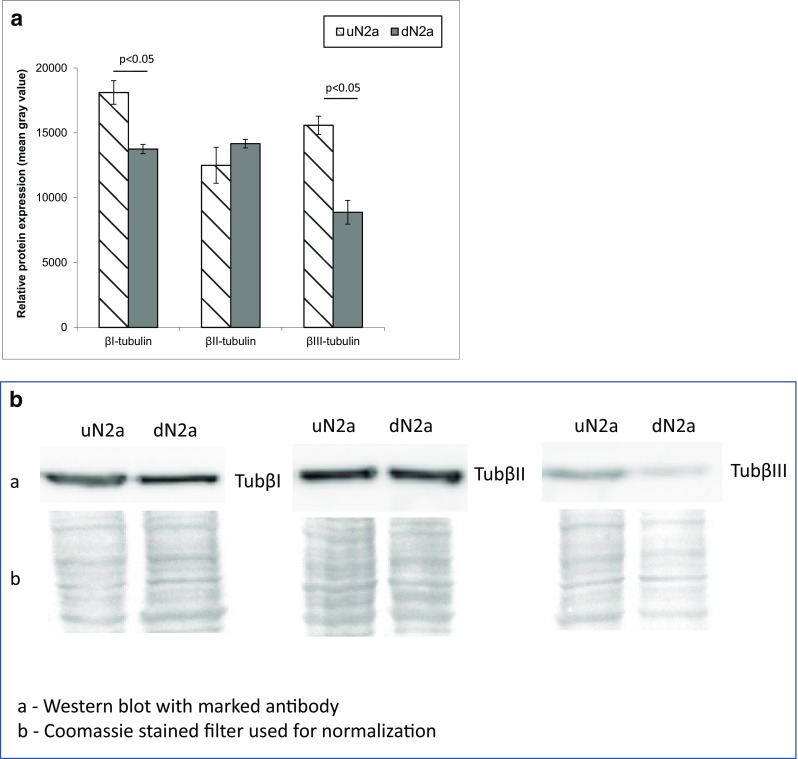
Western blot (WB) analysis of the expression levels of βI-, βII- and βIII-tubulin in undifferentiated and RA-differentiated N2a cells (**a**) as well as the representative WB images (**b**). Error bars are the mean ± SE from 5 independent experiments

**Fig. 4:**
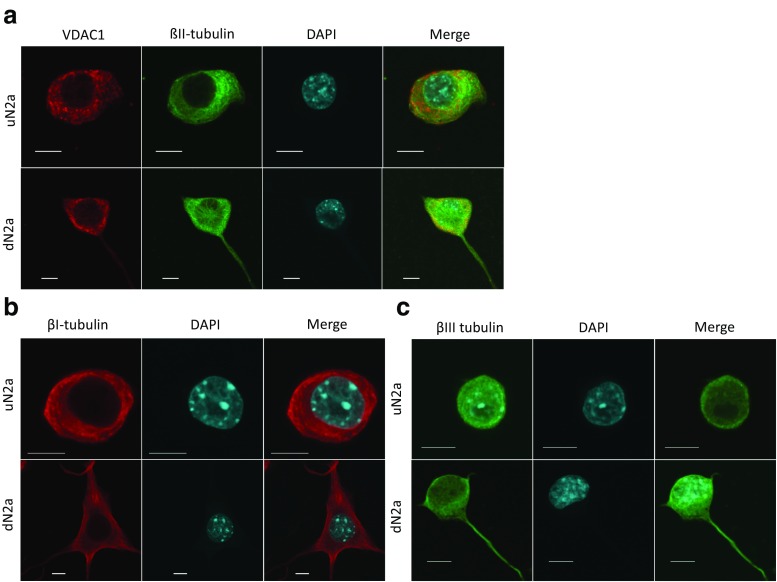
Confocal immunofluorescence imaging of the mitochondrial VDAC1 protein (red), βII-tubulin (green) and their colocalization in undifferentiated (uN2a) and RA-differentiated (dN2a) cells (**a**) distribution of βI- (red) (**b**) and βIII-tubulin isoforms (green) (**c**) in uN2a and dN2a cells. The cell nuclei were stained with DAPI (blue); bars are 10 μm

As βII-tubulin has been shown to regulate MOM permeability in brain synaptosomes, we next characterized the profile of β-tubulin isoforms in cancer cells with neurological origin. N2a cells were maintained in differentiated and non-differentiated states to estimate the alteration of β-tubulin amount and distribution during differentiation. Our results demonstrated that significant changes occurred in the intracellular content of βI- and βIII-tubulin, while βII-tubulin remained at the same level (Fig. 3). Immunofluorescence studies showed that differentiation of N2a was accompanied by remarkable shifts in the intracellular distribution of main β-tubulin isotypes. In uN2a cells, βI-, βII- and βIII-tubulins were localized predominantly around the cell nucleus, whereas in RA-treated cells a part of β-tubulin isotypes were assembled in filamentous structures that crossed the entire cell and neurites (Fig. 4a–c).

### Mitochondrial reserve respiratory capacity in HL-1 sarcoma cells, undifferentiated and differentiated N2a cells

Several works have demonstrated that VDAC gating is regulated by several molecules including glutamate (Gincel et al. 2000), NADH (Zizi et al. 1994) and tubulin (Timohhina et al. 2009). Therefore in the current study we further explored how the availability of main respiratory substrates influences mitochondrial respiration and respiratory reserve capacity. The maximal mitochondrial respiration in the uncoupled state of the respiratory chain was measured by titration of intact HL-1 cells with the mitochondrial uncoupler FCCP in cells growth medium and in medium-B (sees Materials and methods). FCCP is a protonophore that uncouples electron transport and mitochondrial respiration from ATP synthesis by dissipating the proton gradient. We found that high levels of FCCP inhibited mitochondrial respiration in these cells. The FCCP concentration for cells growth medium was 4 μM and for medium-B 2 μM. The mitochondrial reserve capacity was calculated from the V_F_/Vo ratio (Table 1). In HL-1 cells no difference in respiration rate and mitochondrial reserve capacity was seen between growth medium and medium-B. To compare differentiated and undifferentiated N2a cells, both cell cultures were titrated with FCCP in the medium-B. The optimal concentration of FCCP for both cell types was 2 μM. Our results showed that the RA-mediated differentiation of N2a cells increased their mitochondrial capacity in the presence of complex I and II respiration substrates (Table 2). The contribution of complexes I and II to the total mitochondrial reserve capacity was also examined. The complex II activated respiration was measured in the presence of rotenone, an inhibitor of complex I. The addition of rotenone (1 μM) resulted in a 20–30% decrease in the rate of Vo in both uN2a and RA-treated cells (Table 2). Experiments with FCCP suggested that the mitochondrial capacity increased not only through the activation of complex I, but also complex II during the differentiation of N2a cells.

**Table 1:** The effects of FCCP on the respiratory activity of non-permeabilized HL-1 cells

Respiratory medium	Rates of O_2_ consumption, nmol/min per mg protein, mean ± SE, *n* = 6
Claycomb medium^(a)^	^(b)^Vo = 4.9 ± 0.27
	^(c)^V_F_ = 11.34 ± 0.45
	V_F_/Vo = 2.33 ± 0.16
Succ + Mal + Glut^(d)^	Vo = 4.81 ± 0.17
	V_F_ = 12.10 ± 0.03
	V_F_/Vo = 2.52 ± 0.09

^a^full Claycomb medium supplemented with 10% FBS and antibiotics

^b^Vo is the initial rate of O_2_ consumption

^c^V_F_ is the maximal rate of mitochondrial respiration in the presence of 4 μM FCCP

^**d**^in medium-B

**Table 2:** The effects of FCCP on the respiratory activity of non-permeabilized N2a cells (both undifferentiated and RA-treated) in medium-B with/or without the presence of rotenone - an inhibitor of complex-I of the mitochondrial respiratory chain

Respiratory substrates^(a)^	Rates of O_2_ consumption, nmol/min per mg protein, mean ± SE, *n* = 5	
	Undifferentiated N2a cells	Differentiated N2a cells
Mal + Glut	^(b)^Vo = 1.86 ± 0.04	Vo = 1.92 ± 0.1
	V_F_ = 3.03 ± 0.14	V_F_ = 4.21 ± 0.28
	V_F_/Vo = 1.65 ± 0.08	V_F_/Vo = 2.16 ± 0.07; p = 0.005)
Suc + Mal + Glut	Vo = 2.55 ± 0.07	Vo = 2.55 ± 0.12
	V_F_ = 4.65 ± 0.03	V_F_ = 5.81 ± 0.2
	V_F_/Vo = 1.84 ± 0.06	V_F_/Vo = 2.33 ± 0.13; p = 0.01
Suc + Mal + Glut in the presence of Rot	Vo = 2.75 ± 0.25	Vo = 2.8 ± 0.2
	V_R_ = 1.84 ± 0.12 (67%*)	V_R_ = 2.27 ± 0.17 (81.1%*)
	V_F_ = 3.0 ± 0.2	V_F_ = 4.56 ± 0.32
	^(c)^V_F_/V_R_ = 1.65 ± 0.04	V_F_/V_R_ = 2.06 ± 0.09; p = 0.005

^a^Succinate (Suc, at 10 mM), malate (Mal, at 2 mM), and glutamate (Glut, at 5 mM) served as respiratory substrates

^b^Vo is the initial rate of O_2_ consumption

^c^V_F_ and V_R_ are the rates of O_2_ consumption in the presence of 2 μM FCCP and 1 μM rotenone, respectively (rotenone was added 5 min before titrations with FCCP); * % from Vo value

### Mitochondrial contribution to the energy metabolism in HL-1cells with polymerized and unpolymerized tubulin

**Fig. 5:**
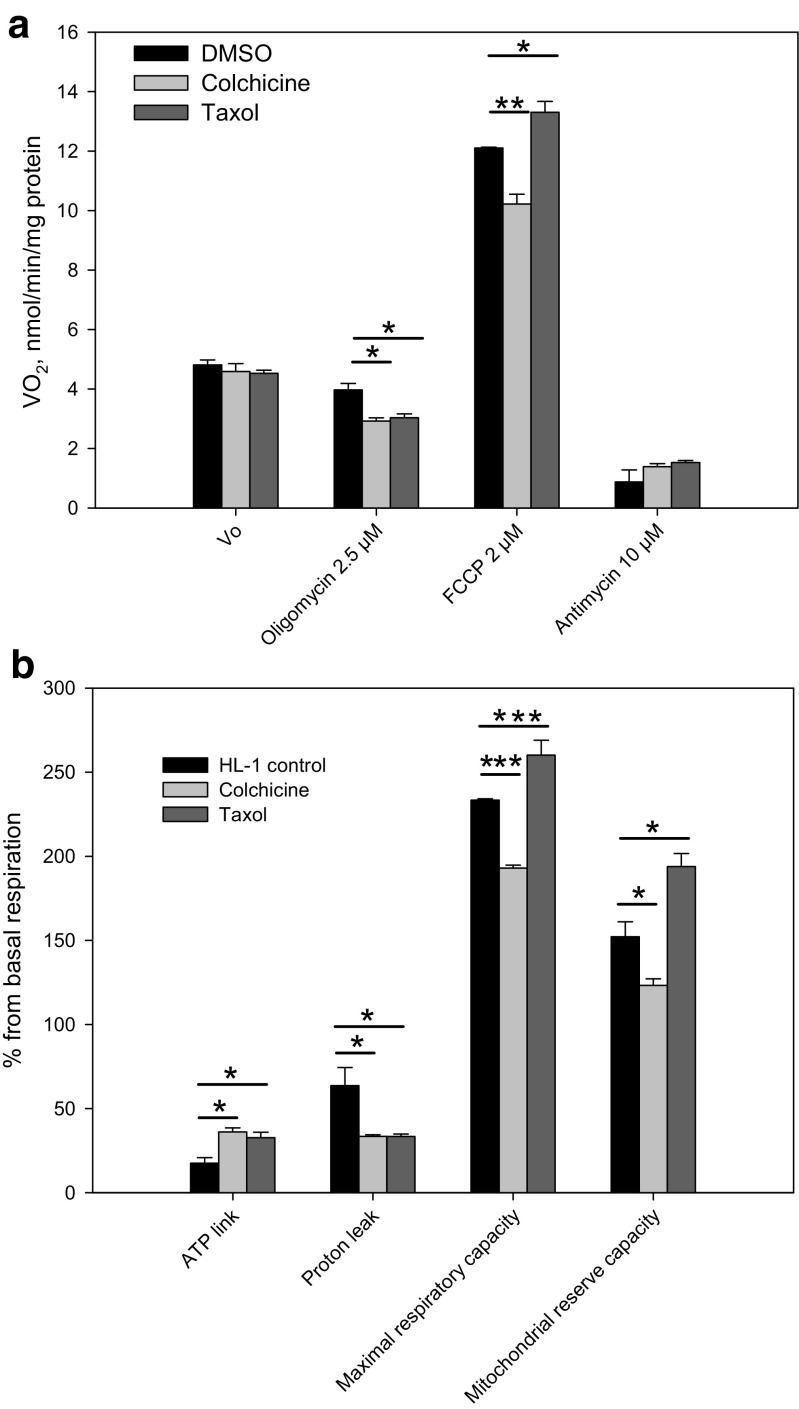
The effects of taxol and colchicine on mitochondrial bioenergetics in HL-1 cardiac tumor cells. **a** Basal respiration – V_0_, responses to treatment with 2.5 μM oligomycin, FCCP and antimycin A. **b** Effects of taxol and colchicine on proton leak, ATP linked respiration, maximal respiratory capacity and mitochondrial respiratory reserve capacity in HL-1 cells compared to control (DMSO treated) cells. Data are shown as mean ± SEM (*n* = 4). Significance stars depict changes in mitochondrial respiration compared to DMSO treated control cells: * p < 0.05, ** *p* < 0.01, *** *p* < 0.001

For understanding the influence of polymerized and unpolymerized tubulin on the mitochondrial contribution of energy metabolism, we analyzed the oxygen consumption rate of the HL-1 cells in the presence of respiratory chain inhibitors (oligomycin, antimycin) and uncoupler of OXPHOS (FCCP) (Fig. 5 a, b). After measurement of basal respiration, the inhibitor of ATP synthase oligomycin was added to uncouple the ATP-linked respiration from the proton leak. The addition of FCCP resulted in an increase of oxygen consumption levels in all samples, but compared to the control, the taxol treatment showed higher response than observed in colchicine treated cells (Fig. 5a). Finally, mitochondrial respiration was inhibited by antimycin. Similar inhibition effects were noticed in drug treated (colchicine, taxol) and untreated HL-1 cells (Fig. 5a). Next, the ATP link, proton leak, maximal respiration capacity and reserve capacity were calculated according to the mitochondrial stress protocol (Supplementary Fig. 3; 4). The mitochondrial stress protocol revealed that both colchicine and taxol treatment increased ATP-linked respiration at the same time decreased proton leak compared to control HL-1 cells (Fig. 5b). However, taxol and colchicine influenced the mitochondrial maximal respiration capacity as well as reserve capacity in different way. On the one hand, taxol increased both mitochondrial capacity parameters, but on the other hand, colchicine decreased them compared to control HL-1 cells (Fig. 5b).

### Mitochondrial contribution to the energy metabolism in HL-1cells with polymerized and unpolymerized tubulin

In neuroblastoma cells colchicine and taxol had no effect on their bioenergetics parameters (Supplementary Fig. 4).

### Analysis of OXPHOS coupling with hexokinase-2

**Fig. 6:**
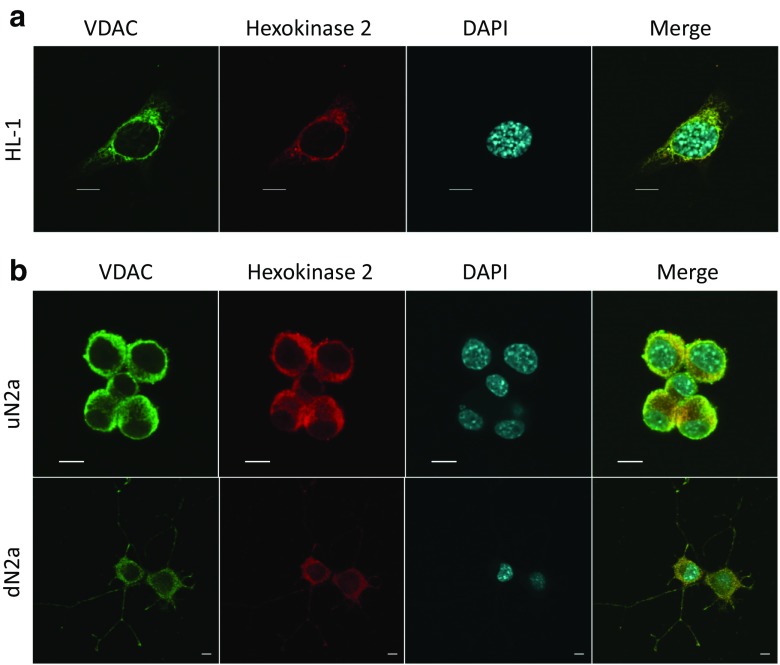
Confocal microscopy imaging of VDAC1 and hexokinase-2 (HK-2) along with their colocalization in HL-1 cells (**a**); and colocalization of VDAC1 with HK-2 in undifferentiated N2a (uN2a) and RA-treated N2a cells (dN2a) (**b**) the cell nucleus (blue, DAPI), HK-2 (red), VDAC1 (green); bars are 10 μM

**Fig. 7:**
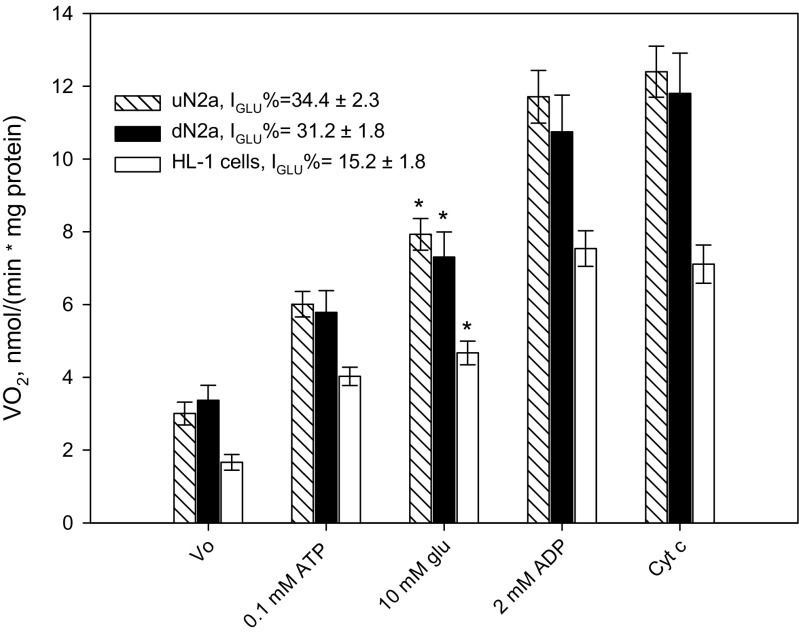
Analysis of the coupling of hexokinase (HK) catalyzed processes with OXPHOS in permeabilized HL-1 cells as well as in undifferentiated (uN2a) and RA-treated N2a cells (dN2a). The efficiency of the coupling between HK and OXPHOS was expressed by the glucose index (I_GLU_). Here: Vo – basal respiration; glu – glucose; and Cyt c – cytochrome c. All data points are the mean from 5 independent experiments; error bars are SEM. *- significant difference, p < 0.05

On the basis of the Pedersen model (Pedersen 2007b), the mechanisms of aerobic glycolysis were examined for HL-1and both N2a cell types. Immunostaining of HL-cells showed clearly the possibility of interactions between VDAC and HK-2 (Pearson’s coefficient = 0.96 ± 0.02; Fig. 6a). The addition of glucose (10 mM) in the presence of ATP resulted in an increase in the rate of O_2_ consumption by these cells, demonstrating thereby the coupling between HK-2 catalyzed reactions and the OXPHOS system, where the strength of functional coupling was quantified by the glucose index (Fig. 7). The same mechanism of aerobic glycolysis was examined for undifferentiated and RA-treated N2a cells. There were no significant differences between respiratory states in these cell cultures. The confocal microscopy of immunostained preparations of undifferentiated and dN2a cells revealed a similar degree for the HK-2-VDAC colocalization (corresponding Pearson’s coefficients were measured as0.83 ± 0.07 and 0.84 ± 0.07, respectively; Fig. 6b). These data, along with the oxygraphic analysis of the functional coupling between HK-2 and OXPHOS (Fig. 7), indicated that differentiation of NB cells had no effect on the binding of HK-2 to VDAC. It is important to emphasize that differentiation of N2a cells has also no effect on the expression of βII-tubulin, a potential competitor for HK-2 for binding sites on the mitochondrial VDAC, in these NB cells (Fig. 3a).

### Rates of maximal respiration and the permeability of mitochondrial outer membrane for ADP in HL-1 and N2a cells

**Fig. 8:**
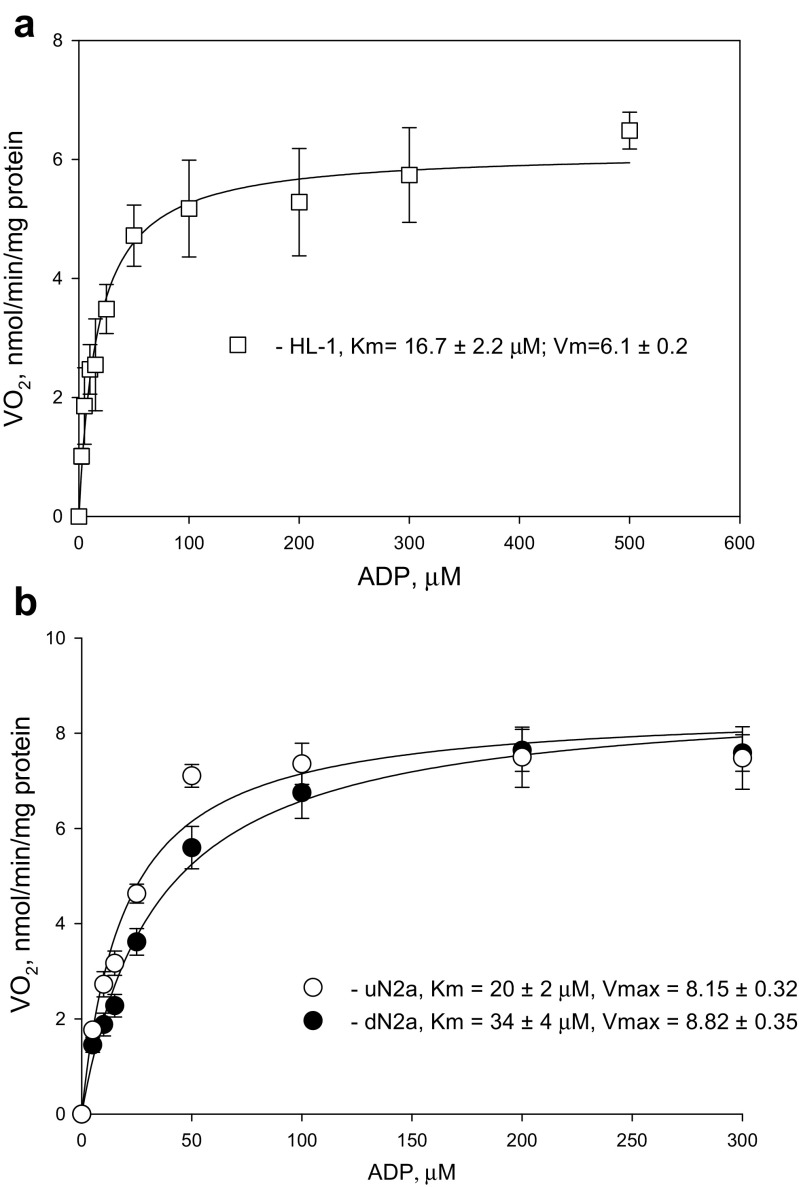
Apparent Km values and rates of maximal (Vm) ADP-activated respiration for HL-1 cells (**a**) as well as for undifferentiated (uN2a) and RA-treated N2a cells (dN2a) (**b**); bars are SEM, *n* = 7

The current study showed the interaction of VDAC with HK-2 in both cardiac sarcoma and N2a cells (Fig. 6). Furthermore, we demonstrated that a big part of total β-tubulin and also βII-tubulin existed as non-polymerized forms (Fig. 1c). Study on N2a cells confirmed that during differentiation towards neuronal cells, the βII-tubulin expression remained at the same level (Fig. 3a). Several studies have indicated that β-tubulin (Maldonado et al. 2010) blocks and HK-2 (Majewski et al. 2004) oppositely keeps VDAC in its open state. Therefore, to clarify the possible role of βII-tubulin and HK-2 in the regulation of VDAC permeability for ADP, tumor cells were titrated with ADP (Fig. 8). Titration experiments showed that the rates of maximal ADP-activated respiration (Vm) were lower in HL-1 cells compared to NB, as well as the Vm did not change during N2a cell differentiation. The affinity of mitochondria for ADP was similarly high in all cells, which indicated that VDAC in tumor cells was in an open state. In addition, treatment of cells with the microtubule destabilizer colchicine, and stabilizer taxol, did not reveal any changes in VDAC permeability for ADP (Table 4).

## Discussion

Recent discoveries in tumor biology propose that targeting of cancer cell energy metabolism can be a novel and effective strategy for suppression of tumor growth and metastasis (Amoedo et al. 2014; Lamb et al. 2014, 2015; Lu et al. 2015). For a long time aerobic glycolysis (Warburg effect) has been considered to be one of the characteristic features of most human cancers (Aminzadeh et al. 2015; Palorini et al. 2014; Pedersen 2007a, b). Several studies have shown that during cancer formation MOM permeability for ADP is altered (Eimre et al. 2008; Kaambre et al. 2012; Kaldma et al. 2014; Klepinin et al. 2014; Maldonado 2017; Maldonado et al. 2010). There are possibly two mechanisms how the MOM permeability for nucleotides is regulated in cancer cells. According to the Warburg-Pedersen model, in cancer cells the HK-2 interacts with VDAC and this interaction results with opened mitochondrial porin channel (Pedersen 2007b). Another mechanism, proposed by Maldonado and co-workers, states that free tubulin and protein kinases dynamically regulate mitochondrial function in cancer cells, but not in untransformed primary cells (Maldonado et al. 2010). Therefore, in the current study we hypothesized that HK-2 and βII-tubulin compete with each other for the VDAC binding site.

Previous studies have shown that the MOM permeability for adenine nucleotides in CM(s) can be regulated through a direct interaction of VDAC with cytoskeletal protein βII-tubulin (Guzun et al. 2012; Varikmaa et al. 2014). Cardiac muscle cells exhibit high apparent Km values (360 ± 51 μM) (Table 3) for exogenously added ADP and this apparent mitochondrial affinity for ADP is not induced by intrinsic Mg^2+^-ATPase activity (Appaix et al. 2003), but controlled by cytoplasmic proteins (Kuznetsov et al. 1996). In the current study, we found that in HL-1 cardiac sarcoma cells most of the cytoskeletal protein tubulin βII was present in the non-polymerized form and some parts of this protein could be associated with MOM (Fig. 1c). Despite this, the permeability of VDAC for ADP in these cells was high and was close to values of those for isolated mitochondria, as well as rat fast-twitch *gastrocnemius w.* muscle cells, where free βII-tubulin was absent (Varikmaa et al. 2014).

**Table 3:** The rates of basal (Vo), maximal (Vm) ADP activated respiration, as well as apparent K_m_ values for ADP for permeabilized adult rat cardiomyocytes (CMs), N2a, and HL-1 tumor cells; these measurements were performed in medium-B with 2 mM malate, 5 mM glutamate and 10 mM succinate, as respiratory substrates

Cells and tissues	Vo	Vm (ADP)	K_m_^app^_ADP_, μM
Rat CM(s)^(a)^	9.3 ± 1	134 ± 6	360 ± 51
Gastrocnemius white, no free βII-tubulin^(c)^	–	–	4.5 ± 1.8
Rat heart mitochondria^(b)^	–	–	17.6 ± 1
HL-1	1.91 ± 0.84	6.1 ± 0.2	16.7 ± 2.2
uN2a	3.38 ± 0.12	8.55 ± 0.32	20 ± 2
dN2a	4.07 ± 0.46	8.82 ± 0.35	34 ± 4
Rat brain synaptosomes^(d)^	–	59 ± 11	110 ± 11
Isolated rat brain mitochondria^(d)^	14 ± 4	36 ± 7	10–20
Brain mitochondria +1 μM tubulin^(d)^	–	–	169 ± 52

All rates of respiration were expressed as nmol O_2_/min/mg protein

^a^from (Anmann et al. 2006; Klepinin et al. 2014)

^b^from (Andrienko et al. 2003)

^c^from (Varikmaa et al. 2014)

^d^from (Monge et al. 2008) 2 mM malate and 5 mM glutamate served as respiratory substrates, and tubulin was in the form of α/β-heterodimer

Taxol give a long-term stability to assembled microtubules, and decrease the free tubulin content inversely to the colchicine, which inhibits microtubule polymerization and increases free tubulin content in cells (Maldonado et al. 2010).). As mentioned above, in neuroblastoma cell culture colchicine and taxol had no effect on their bioenergetics parameters (Supplementary Fig. 4, Table 4). From this, it can be concluded that the role of βII tubulin in mitochondrial energy metabolism of N2a cell culture is small or absent at all. In HL-1 cells colchicine lowered and taxol oppositely raised mitochondrial respiration reserve capacity (Fig. 5b). Our results showed that the mitochondrial respiratory reserve capacity is dependent on the aggregation state of tubulin only in HL-1 cells. The stabilization of microtubules by taxol resulted in increased reserve capacity due to increased maximal respiration. Depolymerization of tubulin, on contrary, decreased respiratory reserve capacity by reducing maximal respiration. Recently, other groups have got similar results on a study on liver cancer cell line HepG2 (Maldonado et al. 2010). They demonstrated that taxol and colchicine not only influenced the cellular free/polymerized tubulin distribution, but also mitochondrial membrane potential. In addition, they found that the increase of free β-tubulin in cancer cells blocked VDAC permeability for nucleotides, and this was the reason why liver cancer tends to aerobic glycolysis. In addition, a study on HepG2 (Maldonado et al. 2010) cells demonstrated that in liver cancer cells the increase of free β-tubulin blocked VDAC permeability for ADP. Nonetheless, in the current study on NB cells and HL-1 cells, such a role of β-tubulin in the regulation of MOM permeability was not observed (Table 4).

**Table 4:** The influence of taxol and colchicine treatment on the affinity of mitochondria for exogenously-added ADP in undifferentiated (uN2a), retinoic acid differentiated (dN2a) and HL-1 tumor cells

Cells and their treatments	K_m_^app^ for ADP, μM ± SEM; n = 4^(a)^	K_m_^app^ for ADP, μM ± SEM; n = 4^(b)^
HL-1 cells, control	16.7 ± 2.2	–
HL-1, colchicine	16 ± 2	–
HL-1, taxol	25 ± 5*	–
uN2a cells, control	20 ± 2	31.7 ± 3.9
uN2a, colchicine	15 ± 2	24.6 ± 3.7
uN2a, taxol	37 ± 3*	30.4 ± 4.5
dN2a, control	–	11.0 ± 0.5
dN2a, colchicine	–	12.3 ± 1.7
dN2a, taxol	–	11.0 ± 1.1

Before respiratory studies these cells were treated for overnight^(a)^ or for 20 min^(b)^ with 10 μM colchicine or 10 μM taxol. Such prolonged (for overnight) treatment of these cells with colchicine and taxol had no effect on the number of viable cells (trypan blue exclusion assay), but was associated with a substantial (~50%) decrease in the rate of their proliferation that was estimated by MTT assay

*- significant difference towards untreated cells; p < 0.05

A possible reason why in HL-1 cells VDACs still remains in an open state, is the interaction with HK-2. Indeed, previously Majewski and co-workers demonstrated that in cancer cells HK interaction with VDAC lead it open for adenine nucleotides (Majewski et al. 2004). Based on the Warburg-Pedersen model we have hereby shown, that in HL-1 cells there exists a tight coupling between HK-2 and OXPHOS (Fig. 6b).

Similar results have been published by another group, where they confirmed, that HK control energy metabolism in these cells (Eimre et al. 2008). The other consequence of the HK–VDAC interaction can result in the prevention of binding of the pro-apoptotic proteins to VDAC, mediating the increased resistance of malignant cells to apoptosis (Pastorino and Hoek 2008).

In the present study we noticed that the maximal rates of ADP-activated mitochondrial respiration did not change during the N2a cell differentiation (Fig. 8b), showing that RA does not influence the quantity of mitochondria; this finding is in good agreement with the recent study performed on human NB cells (Xun et al. 2012). We (see data in Table 2) as well as Xun and colleagues (2012), have demonstrated that RA-induced differentiation increases mitochondrial respiratory reserve capacity in NB cells, which is associated with their metabolism switching from aerobic glycolysis into OXPHOS. Recently Maldonado has hypothesized, that the regulation of MOM permeability for ADP, where free tubulin plays an important role, is the main switch between mitochondrial OXPHOS and glycolysis in malignant cells transformation (Maldonado 2017; Maldonado et al. 2016; Maldonado and Lemasters 2014; Maldonado et al. 2010). For both undifferentiated and differentiated N2a cell lines low apparent Km values for ADP were registered.

The treatment of NB cells with RA also did not increase their mitochondrial respiration rate, the binding of HK-2 to VDAC and its functional coupling with OXPHOS (Figs. 7 and 8b). These results correlate with high affinity of mitochondria for ADP in uN2a and dN2a cells. Altogether, binding of HK-2 with VDAC in both N2a cell lines, as well as in HL-1 sarcoma cells, could mediate their glycolytic phenotype. It has been shown previously, that the total HK activity and the rate of glycolysis of differentiated N2a cells are substantially higher as compared with undifferentiated NB cells (Klepinin et al. 2014; Xun et al. 2012). The reason for this could be the elevated expression of HK-1, which is the predominant isoenzyme in mature neurons (Wilson 2003) and it can also bind to the mitochondrial VDAC (Pastorino and Hoek 2008). Therefore, further studies are needed to clarify the possible contribution of HK-1 to the total glycolytic capacity of NB cells.

The mitochondrial VDAC can be phosphorylated by different serine/threonine kinases in cancer cells, which can regulate the level of the open or closed state of this channel. It has been shown that HK-2 phosphorylation by serine/threonine kinase Akt increases the HK binding to VDAC, which leads to the open state of the channel (Majewski et al. 2004).

It has been reported (Simamura et al. 2008), that cancer cells contain an increased number of VDACs per mitochondrion and, as a result, tumor mitochondria have an enhanced binding capacity for HK-2 compared to normal differentiated cells. In cancer and normal cells HK can only interact with VDAC1 isoform (Anflous-Pharayra et al. 2007; Shoshan-Barmatz et al. 2009). Maldonado and co-workers have reported that VDAC1 is also a binding partner for β-tubulin (Maldonado et al. 2013). Furthermore, they noticed that in HepG2 cells grown in normal conditions, another VDAC isoform VDAC2, was also occupied by β-tubulin and this may lead to the result by which most of VDAC channels stayed in a closed state. According to our previous work most of VDACs stay in CM in the closed state, due to their closure by βII tubulin (Guzun et al. 2011; Varikmaa et al. 2014).

Monge and co-workers have demonstrated that the main role of βII-tubulin is to regulate VDAC permeability for ADP in brain synaptosomes (Monge et al. 2008), but this is not the only function of βII-tubulin in neurons. A silencing study of βII-tubulin in NB cells revealed that βII-tubulin plays an important role in neurite outgrowth (Guo et al. 2010). In the current study, experiments with N2a cells revealed that although the levels of βII-tubulin expression in undifferentiated and RA-treated cells were almost the same, the intracellular localization was different. Olmsted et al. showed, that there are big differences between free and soluble tubulin amounts. Tubulin assembled in differentiated cells was four to five times greater than in nondifferentiated cells, constituting 48–63% and 11–16% of the total tubulin pool in the respective cell types (Olmsted 1981). In uN2a cells βII-tubulin is located around the nucleus, but during differentiation with RA some part of βII-tubulin is incorporated in neurites in these cells (Fig. 4a). In addition, in this study, we established that both class I and III β-tubulin expression is significantly lower in differentiated N2a cells than in non-differentiated cells (Fig. 3). Tubulin βIII (TUBB3) has been reported to be expressed in the mitochondrial membranes (Cicchillitti et al. 2008). It has also been found, that βIII-tubulin is prominently expressed during the fetal and postnatal development of brain (Katsetos et al. 2003). Higher expression levels of βIII-tubulin have been observed in malignancies like gliomas, ovarian and lung cancer cells, in those tumors increased level of βIII-tubulin has been associated with their aggressive behavior and high proliferative rates (Kanojia et al. 2015; Kavallaris 2010; Mariani et al. 2015; McCarroll et al. 2015a; McCarroll et al. 2015b; Parker et al. 2016; Quaas et al. 2015). This isotype also regulates cellular metabolism and glucose stress response signaling to promote cell survival, proliferation in glucose starvation and decreases the reliance of cells on glycolytic metabolism (Parker et al. 2016). This tubulin isoform can be one of the candidates involved in the tubulin dimers, which regulate the mitochondrial outer membrane permeability.

The alternation of MOM permeability for ADP in cancer cells is related to the reorganization of protein supercomplex MI during carcinogens due to the changes in expression of its components (Chekulayev et al. 2015; Chevrollier et al. 2005, 2010; Kaambre et al. 2012; Willers and Cuezva 2011). The regulation of the mitochondrial outer membrane permeability may be related to the presence of post-translational modifications in β-tubulin, participation of other tubulin isoforms, interplay between energy transfer pathways or changes in the phosphorylation state of VDAC channels (Anmann et al. 2014; Rostovtseva and Bezrukov 2012; Sheldon et al. 2011; Tepp et al. 2014; Varikmaa et al. 2014). It has been reported that in adult rat CM(s), which have high Km value for ADP, the spectrum of post-translational modifications of β-tubulin differs substantially from that in HL-1 cardiac sarcoma cells, in which mitochondria have an increased affinity for ADP (Belmadani et al. 2004). Significant differences in the profile of β-tubulin post-translational alterations between mature neurons and NB cells have also been observed (Song and Brady 2015). These alterations could induce a decrease in the capacity of binding of some β-tubulin isotypes to VDAC, and thereby loss of the cytoskeletal protein(s) role in the regulation of the mitochondrial VDAC channel permeability, which is characteristic for some oxidative muscle cells like CMs, m. soleus, and gastrocnemius red (Guzun et al. 2015; Varikmaa et al. 2014) and for mature neural cells (Monge et al. 2008). Moreover, it could be assumed that other β-tubulin or α-tubulin isoforms could also bind to VDAC and influence its conductance (Anmann et al. 2014). At present, the levels and profiles of expression of α-tubulins in malignant cells are totally uncovered and it is also unclear whether tubulin post-translational modifications could influence the interaction of tubulin with VDAC. The permeability of VDAC may be involved in the prevalence of the energy transfer pathway(s). Differences in regulation of VDAC gating between HepG2 and N2a as well as HL-1 cells may be related to the presence of MI key enzyme mitochondrial creatine kinase (MtCK) in HepG2 cells (Uranbileg et al. 2014) and the absence of this enzyme in HL-1 cell culture (Eimre et al. 2008) and N2a cells (Klepinin et al. 2014). Thus, our results show that the regulation of the MOM permeability is more complicated than previously proposed. It has been shown that in some cancers like cardiac (Eimre et al. 2008) and skeletal muscle sarcoma (Patra et al. 2008), neuroblastoma (Klepinin et al. 2014), colorectal cancer (Kaldma et al. 2014) and prostate cancer (Amamoto et al. 2016) MtCK is downregulated. In CM with low permeability of MOM for ADP, was found, that MtCK is tightly coupled with OXPHOS due to the interaction with ANT (Timohhina et al. 2009). In CM and skeletal muscles it has been demonstrated that addition of Cr increases MOM affinity for ADP, but such an effect of Cr on MOM permeability was not observed in glycolytic muscles. This phenomenon may take place due to low expression of MtCK on fast twitching muscles (Varikmaa et al. 2014). Our current and previous studies have shown that in colorectal cancer and cardiac sarcoma cells the apparent Km of ADP is lower as compared to their normal tissues (Eimre et al. 2008; Kaambre et al. 2012; Kaldma et al. 2014). These results correlate with the downregulation of MtCK in those cells (Eimre et al. 2008; Kaldma et al. 2014; Klepinin et al. 2014). A study on MtCK knockout mice confirms this assumption (Kaasik et al. 2001). It has been found that in the MtCK knockout heart muscle the increased permeability of MOM for ADP is 2.5 times. In previous studies on breast cancer and gastric cancer it has been shown that in those cancers MtCK coupled with OXPHOS, and in gastric cancer addition of Cr increased MOM permeability for ADP (Gruno et al. 2006; Kaambre et al. 2012). The interplay between energy transfer pathways, and different binding sites for tubulin and hexokinase to VDAC may be one of the reasons of the high metabolic plasticity of cancer cells, where the selection of metabolic phenotypes leads to growth and invasive advantages.

## Conclusion

The process of the regulation of mitochondrial outer membrane permeability is more complicated and not only based on binding between the VDAC channel and one type of a protein molecule. The current study demonstrates that the presence of mitochondrially bound HK-2 can mediate the “Warburg” behavior of murine NB(s) and cardiac sarcoma cells. Our experiments demonstrated that βII-tubulin plays a minor role in the regulation of energy metabolism in sarcoma cells, in contrast to cardiac and slow-twitch skeletal muscle cells. Based on our results it can also to be concluded, that the binding sites in the composition of MI for tubulin and hexokinase must be different in cancer cells. The alternations in MOM permeability for adenine nucleotides seem to be a characteristic feature of malignant tumors and understanding of this regulation still requires further work.

## Electronic supplementary material

ESM 1 (PPTX 737 kb)

## Abbreviations

ADNs: Adenine nucleotides
AK: Adenylate kinase
BSA: Bovine serum albumin
CM: Cardiomyocyte
CAT: Carboxyatractyloside
CK: Creatine kinase
FCCP: Carbonyl cyanide p-(trifluoro-methoxy)phenyl-hydrazone
HK: Hexokinase
Km: MICHAELIS-Menten constant
NB: Neuroblastoma
N2a: NEURO-2a
uN2a: Undifferentiated N2a cells
dN2a: Differentiated N2a cells
MtCK: Mitochondrial creatine kinase
OXPHOS: Oxidative phosphorylation
MI: Mitochondrial Interactosome
MOM: Mitochondrial outer membrane
PBS: Phosphate buffered saline
PCr: Phosphocreatine
PEP: Phosphoenolpyruvate
PK: Pyruvate kinase
RA: All-trans-retinoic acid
SNS: Sympathetic nerve system
VDAC: Voltage dependent anion channel
Vo: Rate of basal respiration
Vm: Maximal respiration rate
