# Supplementary material for: The complexity of mitochondrial outer membrane permeability and VDAC regulation by associated proteins
Source: J Bioenerg Biomembr. 2018 Jul 12;50(5):339–54. doi: 10.1007/s10863-018-9765-9 (PMC6209068; doi:10.1007/s10863-018-9765-9)
Supplement: Supplementary file 1 — (PPTX 737 kb) [file 10863_2018_9765_MOESM1_ESM.pptx]

## Slide 1
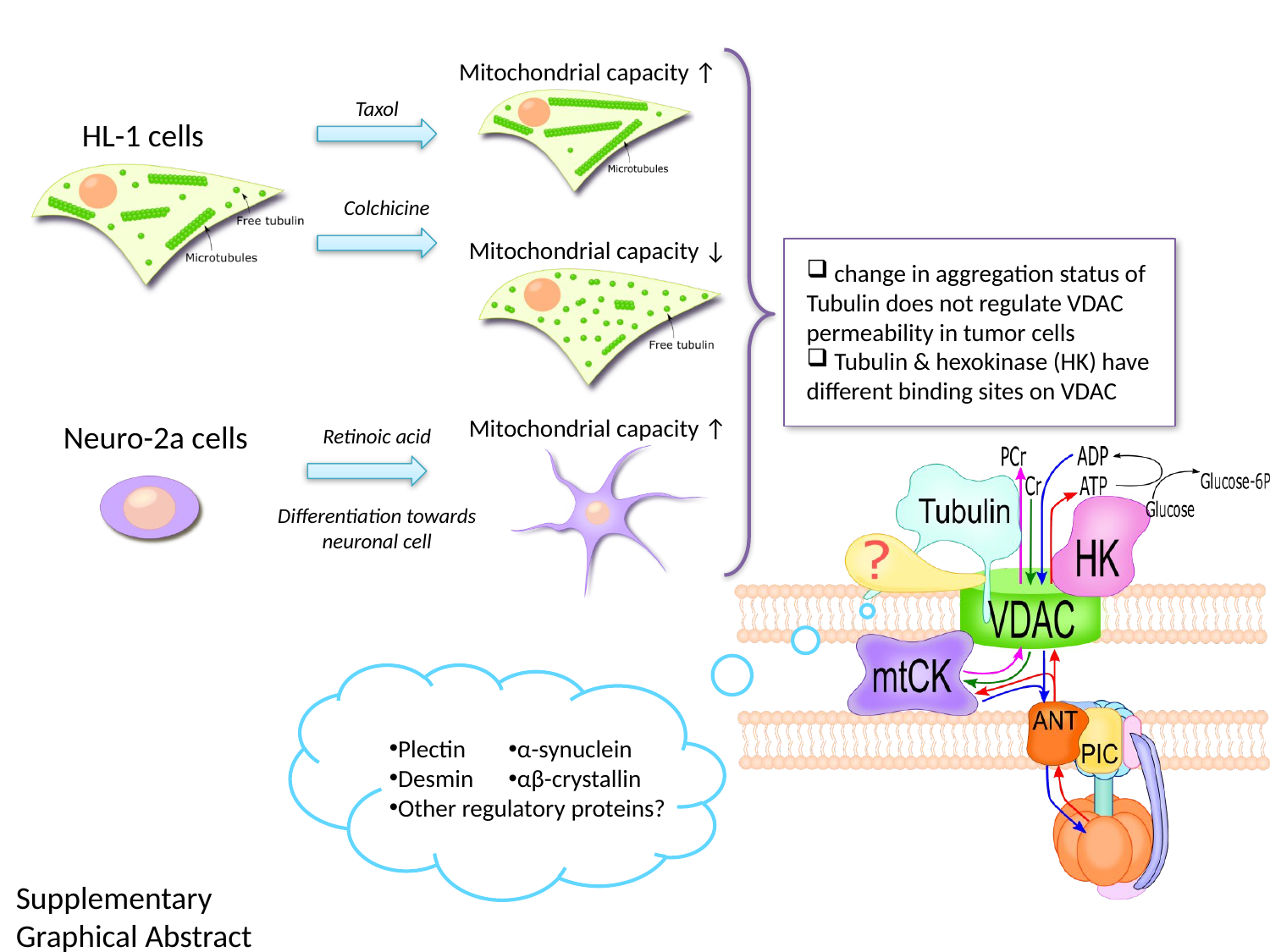

Mitochondrial capacity ↑
Taxol
HL-1 cells
Colchicine
Mitochondrial capacity ↓
 change in aggregation status of Tubulin does not regulate VDAC permeability in tumor cells
 Tubulin & hexokinase (HK) have different binding sites on VDAC
Mitochondrial capacity ↑
Neuro-2a cells
Retinoic acid
Differentiation towards
neuronal cell
Plectin
Desmin
Other regulatory proteins?
α-synuclein
αβ-crystallin
Supplementary Graphical Abstract

## Slide 2
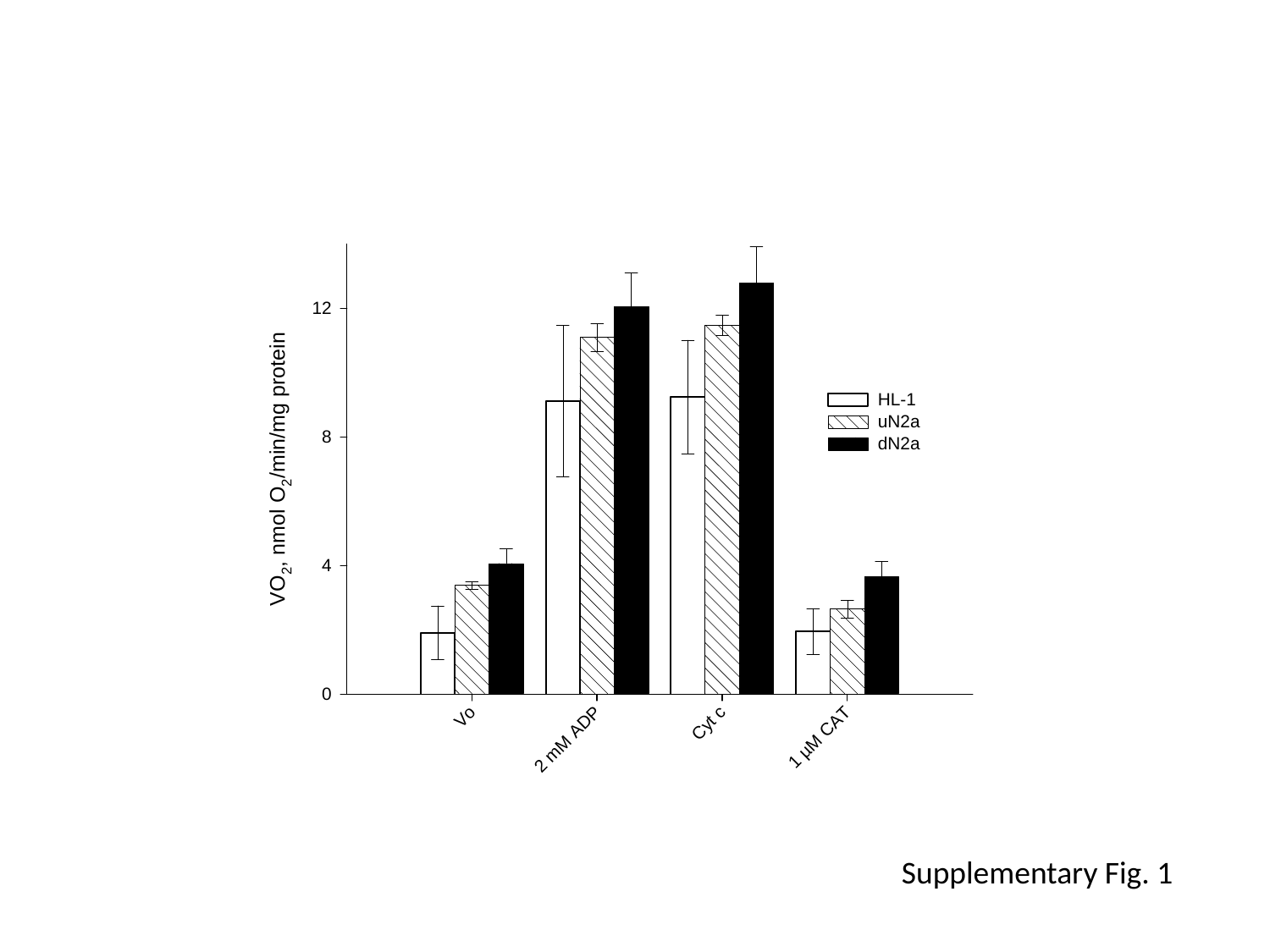

Supplementary Fig. 1

## Slide 3
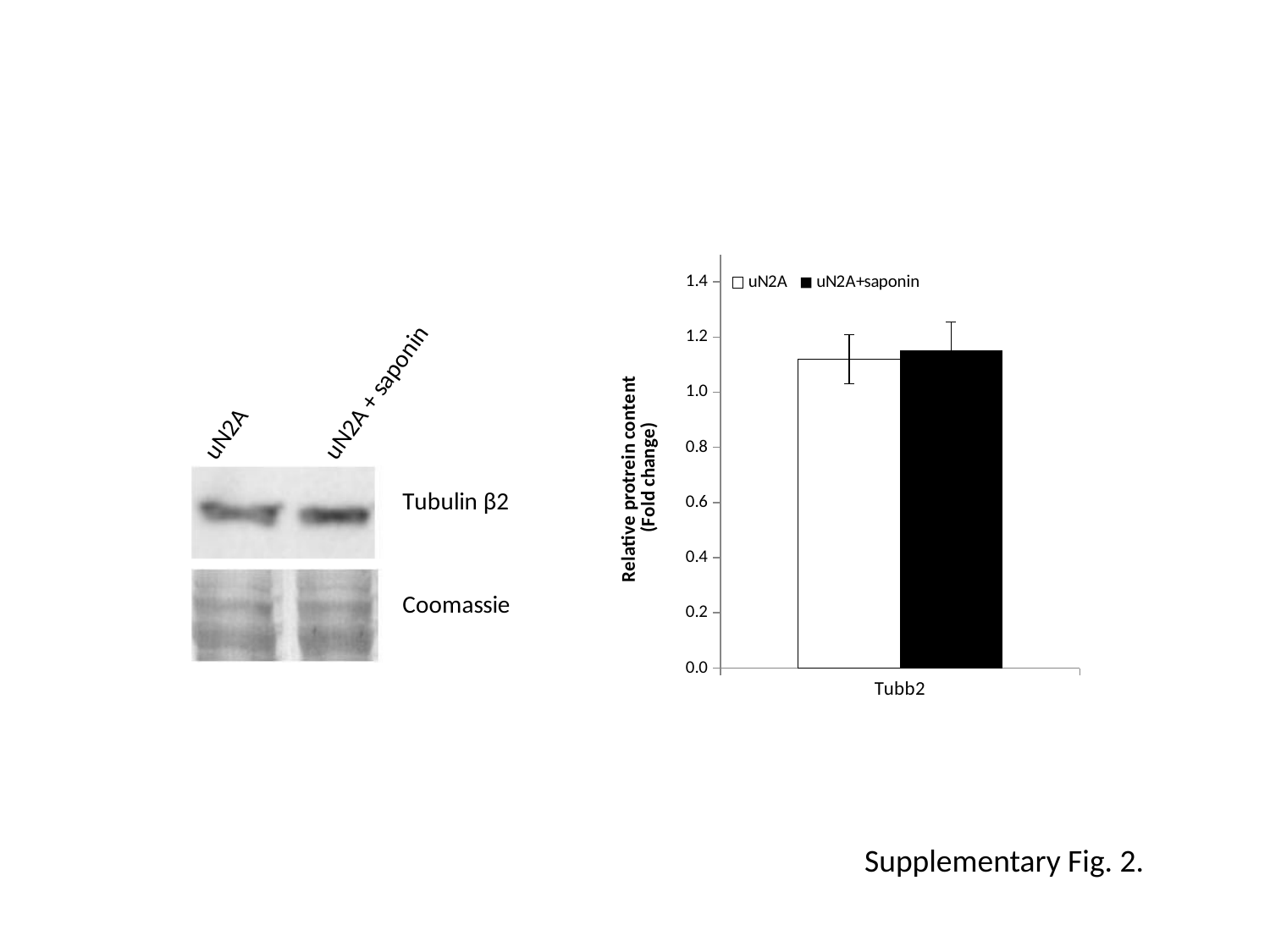

### Chart
| Category | uN2A | uN2A+saponin |
|---|---|---|
| Tubb2 | 1.1198264469989518 | 1.1507178432082303 |Supplementary Fig. 2.

## Slide 4
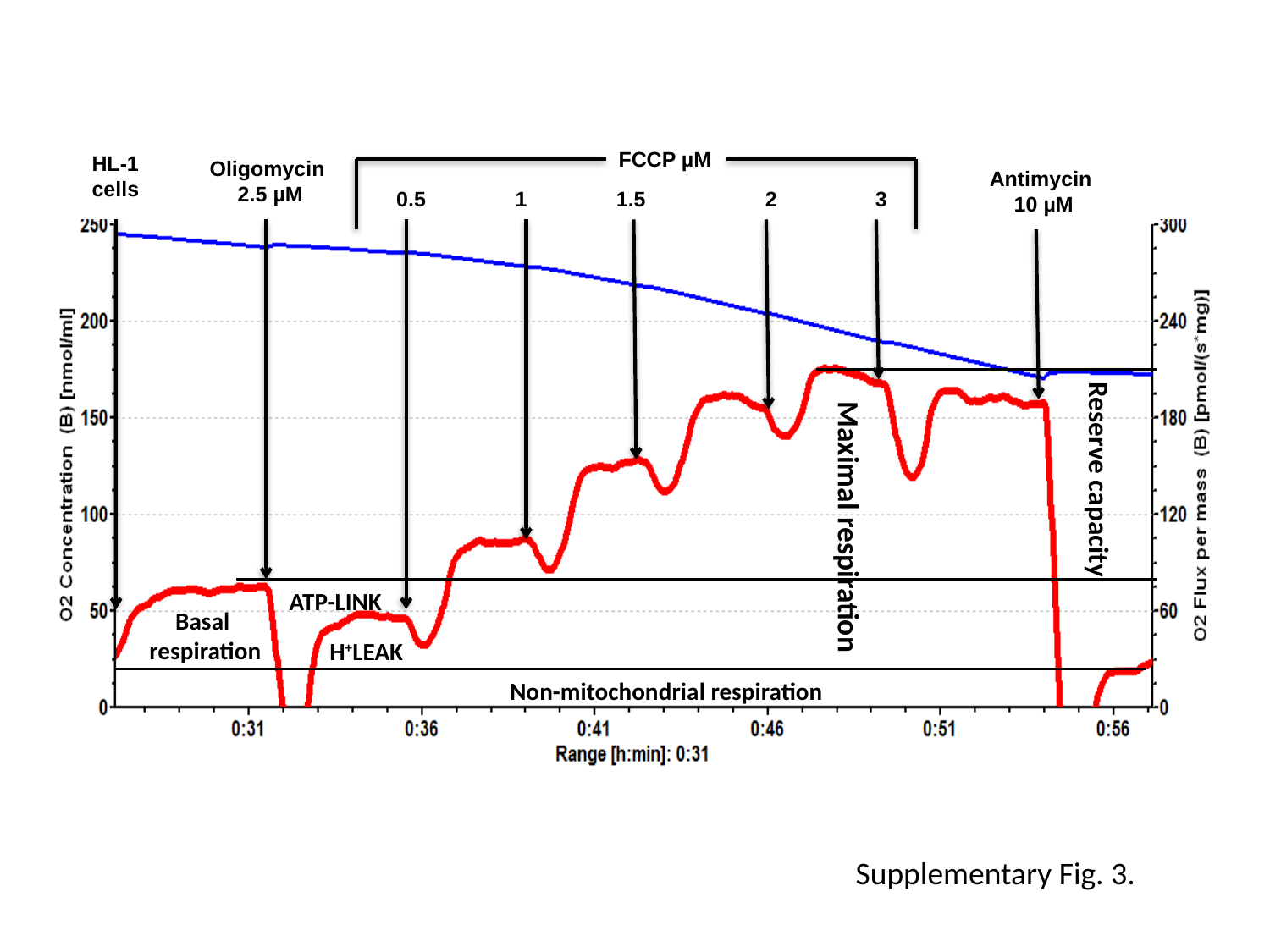

FCCP µM
0.5
1
1.5
2
3
Oligomycin
2.5 µM
HL-1 cells
Antimycin
10 µM
Reserve capacity
Maximal respiration
ATP-LINK
Basal
respiration
H+LEAK
Non-mitochondrial respiration
Supplementary Fig. 3.

## Slide 5
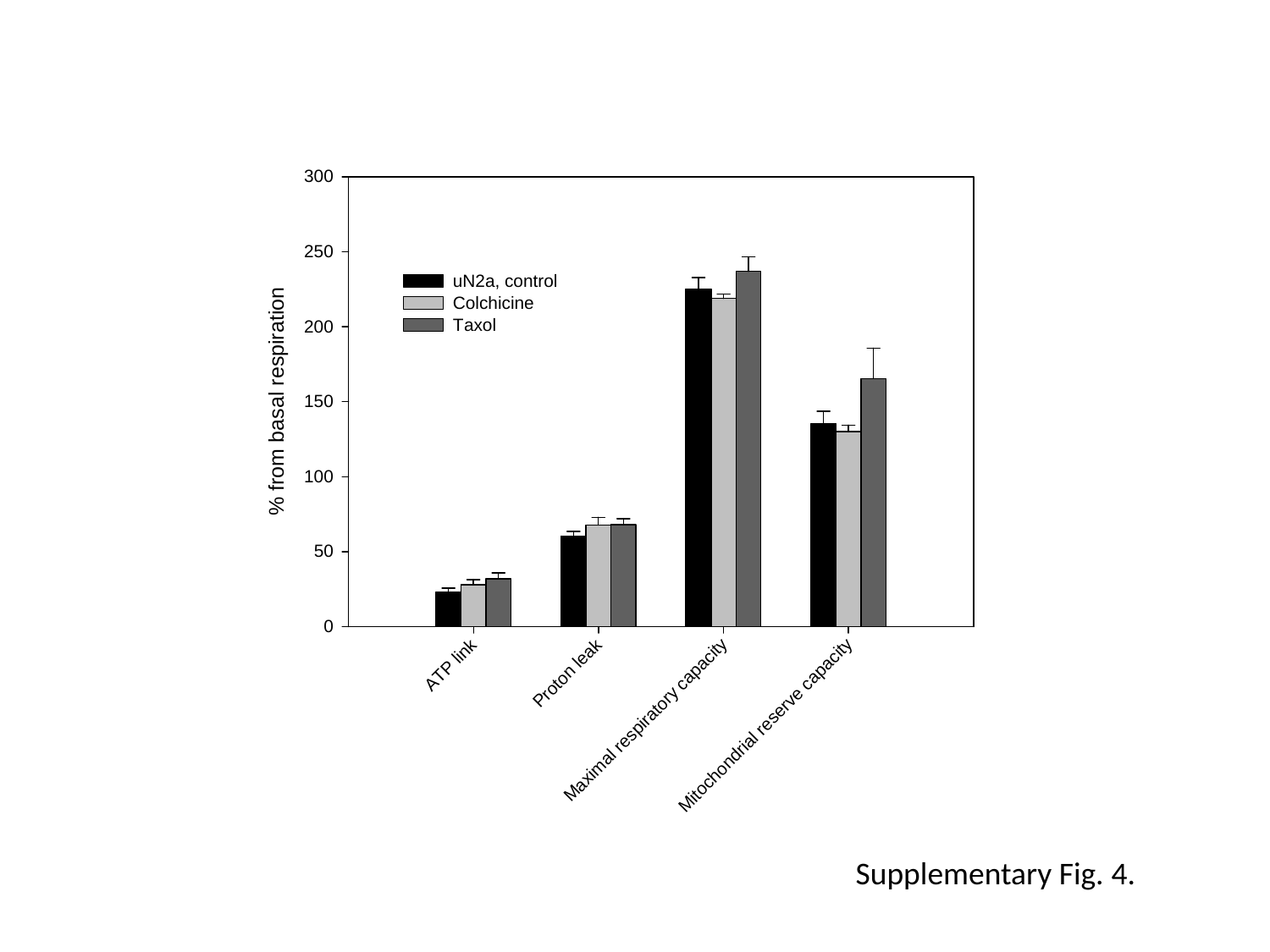

Supplementary Fig. 4.
